# Epidemiology of Meningococcal Disease, New York City, 1989–2000

**DOI:** 10.3201/eid0903.020071

**Published:** 2003-03

**Authors:** Alexandre Sampaio Moura, Ariel Pablos-Méndez, Marcelle Layton, Don Weiss

**Affiliations:** * New York City Department of Health and Mental Hygiene, New York, New York, USA; † Columbia University, New York, New York, USA; ‡ Rockefeller Foundation, New York, New York, USA

**Keywords:** *Neisseria meningitidis*, meningitis, surveillance, serogroup, case fatality rate, New York City, research

## Abstract

Study of the epidemiologic trends in meningococcal disease is important in understanding infection dynamics and developing timely and appropriate public health interventions. We studied surveillance data from the New York City Department of Health and Mental Hygiene, which showed that during 1989–2000 a decrease occurred in both the proportion of patients with serogroup B infection (from 28% to 13% of reported cases; p<0.01) and the rate of serogroup B infection (from 0.25/100,000 to 0.08/100,000; p<0.01). We also noted an increased proportion (from 3% to 39%; p<0.01) and rate of serogroup Y infection (from 0.02/100,000 to 0.23/100,000; p<0.01). Median patient age increased (from 15 to 30 years; p<0.01). The case-fatality rate for the period was 17%. As more effective meningococcal vaccines become available, recommendations for their use in nonepidemic settings should consider current epidemiologic trends, particularly changes in age and serogroup distribution of meningococcal infections.

Meningococcal disease is a broad term used to describe the different clinical syndromes resulting from *Neisseria meningitidis* infection. Its two major clinical illnesses, meningitis and meningococcemia (i.e., sepsis caused by meningococcal infection), occur more often as sporadic cases, but occasional outbreaks are an important cause of illness and death worldwide.

In the United States, a substantial proportion of cases of meningitis and sepsis are caused by *N. meningitidis* (*1*). The incidence rate of meningococcal disease in the United States is estimated to be 0.7–1.4/100,000 population, and the case-fatality rate (CFR) is approximately 10% (*2*,*3*). Both the incidence rate and CFR have been relatively constant, with no major changes observed in the past decade (*2*).

Serogroups B and C are the most common strains found in the United States; however, increased rates of infection from serogroup Y were observed in the 1990s (*2*,*3*). Changes in the age distribution of those infected have also been noted, and the conventional concept that meningococcal disease predominately affects infants and young children should be revised because the median age of meningococcal disease case-patients has increased (*2*).

We describe the epidemiology of meningococcal disease in New York City from January 1989 to December 2000 with an emphasis on the trends of serogroup incidence, age, and fatality rates. Results confirmed the following hypotheses: Consistent with the trends in the epidemiology of meningococcal disease in the United States, the incidence of serogroup Y infection in New York City is increasing, the median age of patients is increasing, and the CFR is comparable to national figures.

## Methods

The study included New York City residents who met the case definition for confirmed or probable meningococcal disease, as defined by the Centers for Disease Control and Prevention and the Council of State and Territorial Epidemiologists. Inclusion in the study as a confirmed case required a clinically compatible course with either the isolation of *N. meningitidis* from a sterile site (e.g., blood, cerebrospinal fluid, joint fluid, or pleural fluid); inclusion as a probable case required a positive antigen test from cerebrospinal fluid or clinically described purpura fulminans (*4*). The period of study was January 1989–December 2000.

We obtained the meningococcal disease cases from the New York City Department of Health and Mental Hygiene (referred to hereafter as NYC Department of Health) surveillance database of reportable diseases. Meningococcal disease is a national reportable disease; in New York City, all cases are required by health code to be reported to the NYC Department of Health. Physician reports, investigation forms, and laboratory reports were reviewed for all the meningococcal disease patients included in the NYC Department of Health database. All cases with evidence of the study criteria were included. Data on meningococcal disease used in this study were collected through routine passive surveillance, and serogroup identification was performed by the NYC Department of Health Public Health Laboratory. Antibiotic resistance profiles and pulsed-field gel electrophoresis results were available only for a subset of isolates after 1999 and are not included in this report. Archival data and population estimates (before 1989) were obtained from New York City Vital Statistics Annual Summary reports.

The database contained information on each cast-patient’s age, sex, race, ethnicity, borough of residence, and death. Information on race and ethnicity was incomplete and therefore was not analyzed. When death information was missing, patient identifiers were submitted to the New York City Vital Records and Registry for a death certificate search, which was accomplished by searching by name and International Classification of Diseases (ICD) code. Using name search, staff in the New York City Vital Records and Registry department used visual inspection to search the New York City death certificates, looking for the name of each patient with an unknown cause of death in the 1-month period after the date of onset of the disease. We also conducted a search using the ICD codes that correspond to meningococcal disease (ICD-9 036.0–036.9 and ICD-10 A39.0–A39.9); the search identified all death certificates from 1989 to 2001 that included these codes. We used the information found through this second search method if the death certificate referred to a patient already in the database with an unknown outcome. We did not include death certificates with meningococcal disease ICD codes that referred to patients not previously included in the database (i.e., they had not been reported to the NYC Department of Health as having meningococcal disease) because of the lack of data to confirm the diagnosis. Patients whose names did not appear in the death certificate search file were considered survivors in the CFR calculation. This study was based on electronic data and surveillance records; we ensured confidentiality by excluding all identifying information from the active analysis database.

### Statistical Methods

Incidence rates were calculated by using 1990 and 2000 population files from the U.S. Census Bureau. We used the Pearson chi-square test or Fisher exact test to assess the statistical significance of categorical variables and the Kruskal-Wallis test to assess continuous variables.

Time trend analysis was performed to detect an association between time (e.g., year or year group) and response variables (e.g., serogroup and outcome). We used the Spearman correlation test and chi-square test for linear trends to assess statistical significance. Logistic regression models were built to provide coefficients for significant trends.

Independence can be assumed from the data because most cases were sporadic throughout the study period; we considered the vast majority of cases to be unrelated. In addition, no patient had more than one episode of the disease during the study period, and the analyses were performed with the patients grouped into 3-year intervals to minimize any existing correlation between sequential years (*5*). The SPSS (SPSS Inc., Chicago, IL) statistical software package and Epi Info 2000 (Centers for Disease Control and Prevention, Atlanta, GA) software were used to perform the statistical calculations.

## Results

Among New York City residents, 615 cases of meningococcal disease were reported to the NYC Department of Health from January 1989 to December 2000, with an average annual incidence of 0.67/100,000; of cases reported, 582 cases (95%) were confirmed and 33 cases (5%) were included as probable. Meningococcemia occurred in 54% of the cases, meningitis in 44%, and pneumonia, septic arthritis or other sterile site infections in 2%. All cases were considered to be sporadic except for two case-patients in 1997 who were contacts of a primary case-patient in a juvenile detention center resident and one culture-negative case-patient in 2000 who was linked to a subsequent confirmed case-patient by household contact.

For the period 1989–2000, the meningococcal disease rate decreased by 33%, compared to the period 1953–1988, and declined by 90%, compared to the period 1905–1952 (Table 1). During the period under study, a 69% reduction occurred at the beginning of the 1990s, with the rates dropping from 1.19 per 100,000 population in 1989 to 0.37 per 100,000 population in 1992 (chi square for trend = 9.1; p<0.01). Since then, rates have increased slightly and remained relatively constant (Table 1). When children <1 year of age are excluded, the declining trend in incidence is no longer statistically significant (chi square for trend = 2.4; p<0.12).

**Table 1:** Rates of meningococcal disease, New York City, 1905–2000

Yr group or yr	Cases^a^	Annual rate/100,000
1905–1916	7,038	12.3
1917–1928	3,715	5.44
1929–1940	3,844	4.29
1941–1952	4,505	4.75
1953–1964	1,007	1.08
1965–1976	707	0.75
1977–1988	986	1.16
1989	87	1.19
1990	79	1.08
1991	30	0.41
1992	27	0.37
1993	40	0.55
1994	42	0.57
1995	54	0.73
1996	59	0.80
1997	54	0.73
1998	35	0.47
1999	59	0.79
2000	50	0.62
1989–2000	615	0.67

^a^Before 1945, meningococcal disease was classified as cerebrospinal fever or epidemic spinal meningitis.

When stratified by borough of patient residence, the average incidence rates were highest in the Bronx (0.88/100,000) and Manhattan (0.81/100,000) and lowest in Brooklyn (0.65/100,000), Staten Island (0.65/100,000), and Queens (0.55/100,000). However, the differences between boroughs were not statistically significant (chi square = 1.4; df=4; p=0.23). Rates by United Hospital Fund neighborhoods ranged from 0.23 to 1.08 per 100,000. The highest rates occurred in two northern Manhattan and one central Bronx neighborhoods; the lowest rates were all in Queens.

The highest average annual incidence rate was observed among patients <1 year of age (8.49/100,000), with substantially lower rates observed for older age groups (Table 2). A statistically significant declining trend for the age groups of <1 years of age (chi square for trend **=** 21.5; p<0.01) and 1–4 years of age (chi square for trend **=**14.3; p<0.01) was seen over the four 3-year groups. No other decrease or increase in age-specific incidence trends was statistically significant. The proportion of cases occurring in young children (<5 years of age) decreased from 39% in 1989–1991 to 17% in 1998–2000.

**Table 2:** Meningococcal incidence rates and case-fatality rates by age group and year group, New York City, 1989–2000^a^

Age group (yr)	1989–1991	1992–1994	1995–1997	1998–2000	1989–2000	Case-fatality rate (%)
<1	15.9	8.15	7.25	4.23	8.49	13.0
1–4	2.83	1.10	1.24	0.85	1.50	13.0
5–14	0.77	0.37	0.61	0.40	0.53	7.8
15–24	0.99	0.76	0.45	0.72	0.77	10.6
25–44	0.34	0.21	0.49	0.47	0.38	17.1
45–64	0.47	0.26	0.61	0.55	0.48	24.4
>65	0.77	0.45	0.75	0.60	0.64	32.9
All ages	0.89	0.50	0.69	0.60	0.67	16.9

^a^Rates are per 100,000.

The overall median age of the patients with meningococcal disease was 22 years; stratification of diagnosis by year group showed that median age has increased from 15 years of age in 1989–1991 to 30 years of age in 1998–2000 (Kruskal-Wallis test; chi square = 20.0; df =3; p<0.01). To assess the effect of changes in serogroup on the median age, serogroups B, C, Y, and unknown were sequentially excluded from the computation of median age. Only the removal of serogroup Y resulted in a loss of statistical significance of the trend in median age (Kruskal-Wallis test; chi square = 7.6; df=3; p=0.06).

Overall incidence rates were higher for males (0.73/100,000) than females (0.61/100,000; relative risk [RR] = 1.19; 95% confidence interval [CI] 1.02 to 1.40). However, CFR was higher (20.1% vs. 13.9%; RR=1.45; 95% CI 1.02 to 2.07) for females. No statistically significant differences were found in gender-specific incidence rate by age category.

Serogroup was determined for 423 (72%) of 582 culture-positive cases. From 1989 to 2000, serogroups B, Y, and C were the most commonly identified serogroups (32% [n=137], 28% [n=119], and 27% [n=112], respectively) of the cases for which a serogroup was known. Serogroup W135 constituted 7% (n=28); nongroupable, 3% (n=13); A, 2% (n=9); and other serogroups, 1% (n=5) of the isolates. The median age of the case-patients differed by serogroup, with the highest median age for nongroupable (48 years of age), followed by other (43 years of age), Y (37 years of age), A (34 years of age), W (27 years of age), C (23 years of age), and B (11 years of age).

Incidence rates for serogroup B infections declined in all age groups with the largest decline in the <1-year and 1–4-year age groups in 1989–2000. Serogroup Y incidence rates increased twofold to tenfold in all age groups except 1–4 years during the period (Table 3).

**Table 3:** Annual incidence rates of *Neisseria meningitidis*, serogroups B and Y, New York City, 1989–2000^a^

Age group (yr)	1989–1991				1992–1994				1995–1997				1998–2000			
	B		Y		B		Y		B		Y		B		Y	
	No.	Rate	No.	Rate	No.	Rate	No.	Rate	No.	Rate	No.	Rate	No.	Rate	No.	Rate
<1	15	5.8	2	0.78	11	4.3	1	0.39	9	2.7	3	0.91	4	1.2	5	1.5
1–4	13	1.0	0	0	5	0.39	2	0.16	4	0.31	2	0.15	2	0.15	1	0.08
5–14	3	0.11	1	0.04	4	0.15	0	0	3	0.09	3	0.09	1	0.03	9	0.27
15–24	9	0.29	1	0.03	5	0.16	4	0.13	2	0.06	3	0.09	3	0.09	7	0.21
24–44	6	0.08	1	0.01	4	0.05	1	0.01	8	0.10	15	0.19	3	0.04	7	0.09
45–64	5	0.12	0	0	1	0.02	2	0.04	2	0.04	11	0.22	4	0.08	17	0.33
≥65	3	0.10	0	0	4	0.14	3	0.10	2	0.07	8	0.28	2	0.07	10	0.36
All ages	54	0.25	5	0.02	34	0.15	13	0.06	30	0.12	45	0.19	19	0.08	56	0.23

^a^Rates are per 100,000 and use 1990 and 2000 census figures.

**Figure 1:**
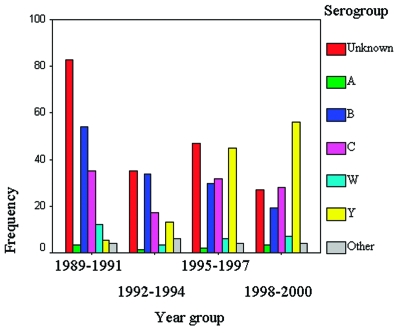
Distribution of meningococcal serogroups by year group, New York City, 1989–2000.

Over the 12-year interval, the proportion of cases caused by strains included in the quadrivalent vaccine available in the United States (A, C, Y, and W135) increased from 28% to 65% of reported cases (Kruskal-Wallis test; chi square = 57.4; df=3; p< 0.01). This increase is due in part to the decline in incidence of serogroup B infections and the decline in the number of cases for which a serogroup could not be determined (Figure 1).

To assess whether changes in serogroup B and Y incidence were independent from the changes observed in age, we performed logistic regression analyses. The likelihood of serogroup Y infection compared with all other serogroups increased by a factor of 2.47 (99% CI 1.84 to 3.33) for each successive year group while controlling for age. The likelihood of serogroup B infections compared with all other serogroups decreased by a factor of 0.77 (99% CI 0.61 to 0.91) for each successive year group.

Information about patient outcome was initially available for 478 (77.7%) of the cases. After the vital records search, one additional death was identified for a patient with missing outcome. The overall CFR during 1989–2000 was 16.9% (104 deaths, 615 cases). The CFR varied during the study period, being lowest in the interval 1992–1994 (14%; 15/109) and highest in 1989–1991 when 20% (39/196) of the case-patients died; however, the difference between year groups was not statistically significant. When we analyzed CFR for each year separately, we found a surprisingly high CFR of 27% (16/59 cases) in 1999.

**Figure 2:**
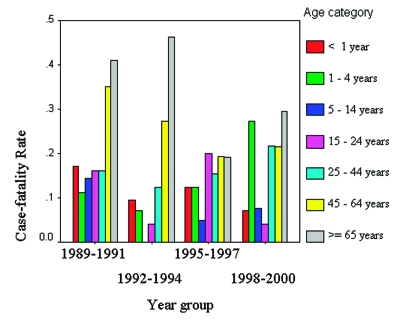
Meningococcal case-fatality rate by age category and year group, New York City, 1989–2000.

CFR increased linearly with age after 5 years of age and was lowest for those 5–14 years of age (8%) and highest for >65 years of age (33%) (Table 2). Figure 2 shows CFR by age category and year group. The CFR also differed by serogroup and was the highest for serogroup A (44.4%; 4/9), compared to that observed among serogroups C (22.3%; 25/112), Y (18.5%; 22/119), W (17.9%; 5/28), and B (12.4%; 17/137). However, the high CFR for serogroup A should be interpreted cautiously because of the low number of cases in the study period. No statistically significant difference of CFR between serogroups was noted (Bonferoni adjustment for multiple comparisons, p>0.002).

## Discussion

Our study has shown that a significant decrease in meningococcal disease incidence rates occurred at the beginning of the 1990s in New York City, and low incidence rates were observed throughout the rest of the decade. The decline in incidence rates are unlikely to have occurred because of changes in surveillance; no modifications in the diagnostic criteria for meningococcal disease were made, and only passive surveillance was conducted throughout the entire study period.

Compared to national surveillance data, the overall incidence rate in New York City during the period was 34% lower (0.67 vs. 1.02/100,000) (*6*). Age-specific rates in children <5 years of age have declined both nationally and in New York City, although the magnitude of the decline in the city has been greater. Nationally, the rate for patients <1 year of age declined from 13.5/100,000 in 1989 to 6.79/100,000 in 2000. For children 1–4 years of age, the rate declined from 4.18/100,000 to 2.04/100,000 over the same period (*6*). Much of the decline occurred during the years 1998–2000. For New York City, the rates in the <1-year age group declined from 18.6/100,000 in 1989 to 2.72/100,000 in 2000. For the 1–4 year age group, the rate declined from 4.95/100,000 in 1989 to 0.93/100,000 in 2000. A similar trend for New York State (excluding New York City) has occurred, with overall meningococcal rates dropping 44% (from 1.28/100,000 in 1989 to 0.72/100,000 in 2000) and the rate <5 years declining 85% (from 8.85/100,000 to 1.29/100,000, unpub. data, New York State Department of Health, Division of Epidemiology).

The median age of New York City case-patients was higher than that observed in epidemiologic reviews for the United States and the New England region (*2*,*6*). The increase in the median age of cases from 1989 to 2000 observed in New York City is predominately due to a decrease in the incidence rates among young children (1–4 years of age) and infants (<1 year of age), along with slight increases in rates among adults (25–64 years of age). This finding of higher median age may be a result of the greater decline in meningococcal disease seen in children <5 years of age in New York City compared to the rest of the United States.

In accordance with trends observed in other areas of the United States (*2*,*3*), a significant increase in the incidence of serogroup Y infection occurred in New York City. The median age of patients with serogroup Y infections in New York City (30 years) was comparable to that of Connecticut patients (29 years) but greater than that seen in Illinois patients (16 years) when comparison data from 1989–1996 were used (*3*). Serogroup C was responsible for an increasing number of sporadic cases and outbreaks in the United States (*7*) and Canada (*8*) in the late 1980s and early 1990s. In New York City, the single cluster involving three people in 1997 was caused by serogroup C infection. The incidence of serogroup C infection did not change substantially in New York City throughout the 12 years of the study; serotype C infection accounted for 18.2% of the total number of cases (range per year group 15.6–19.4).

Serogroup W-135, important worldwide because of the cases associated with returning pilgrims from Saudi Arabia, accounted for 4.6% of all cases in New York City from 1989 to 2000. This association with the pilgrimage to Mecca accounted for the three cases in New York City caused by serogroup W-135 reported from January to April in 2000: One patient was a returning pilgrim, another was a household contact of a returning pilgrim, and the third patient reported having interacted with returning pilgrims or their families (*9*).

The incidence rate of serogroup B declined threefold during the period of study, and the infection has nearly disappeared in children <5 years of age in New York City (one case in 1999–2001). The number of serogroup B meningococcal cases in New York State (excluding New York City) has also declined, from 10 in 1997 to 3 in 2000 (unpub. data, New York State Department of Health, Division of Epidemiology). Data from New Jersey indicate that the number of meningococcal infections in children <5 years of age has similarly declined, from 16 in 1995 to 3 in 2000, although the number of serogroup B cases has remained relatively constant (unpub. data, New Jersey Department of Health and Human Services). In Oregon, where an increase in serogroup B meningococcal disease occurred in the last decade (*10*), no similar decline in serogroup B has been seen in children <5 years of age (Frederick Hoesly, pers. comm.). An interesting discovery is the coincident increase in the use of *Haemophilus influenzae* conjugate vaccine containing serogroup B meningococcal outer membrane protein. Private provider vaccine orders received by the Vaccine for Children program for NYC indicate that the proportion of *H. influenzae* vaccine containing serogroup B meningococcal outer membrane protein has risen steadily from 0% in 1994 to 52% in 2000 (unpub. data, Department of Health, Immunization Program). Comparison Vaccine for Children data for the public sector are incomplete. Studies conducted by the manufacturer found that immunity to serogroup B meningococcus was induced by the serogroup B meningococcal outer membrane protein vaccine in a primate animal model (*11*) and in children during phase III vaccine trials (Alan Shaw, pers. comm.). Further epidemiologic and immunologic research are needed to explore the protective immunity and potential use of this vaccine for meningococcal serogroup B disease.

A significant change occurred in the prevalence of vaccine-preventable strains during the period. During the years 1989–1991, only 29.7% (43/145) of the meningococcal infections that occurred in patients >2 years of age were caused by vaccine-preventable strains. The proportion of vaccine-preventable strains increased steadily in each 3-year interval reaching 66.4% (85/128) in 1998–2000 (chi square for trend; p<0.01). Currently, the quadrivalent meningococcal polysaccharide vaccine, the only licensed and approved vaccine in the United States, provides good efficacy against serogroups A, C, W-135, and Y infections in older children and adults. The vaccine is not routinely recommended for the general population because of its short duration of protection, poor efficacy in children <2 years of age, and the low incidence of meningococcal infections in the United States (*1*,*12*). To overcome the problems of immunity in young children, conjugate vaccines have been recently developed and might dramatically improve the prevention of meningococcal disease because of their greater efficacy among infants and longer duration of immunity (*13*). The conjugate vaccine that is currently licensed in the United Kingdom only protects against serogroup C infection; its addition to the routine childhood vaccine schedule in New York City would have limited impact based on current serogroup incidence (*13*). Meningococcal serogroup C accounted for 12% (3/25) of infections in children <5 years of age and 2% (3/144) of all meningococcal infections in 1998–2000. During the entire 12-year period, only one serogroup A meningococcal infection occurred in a child <5 years of age. To make an impact on rates of meningococcal disease in New York City through routine childhood vaccination, a vaccine is needed that produces good, long-lasting immunity in young children to serogroups B, C, W-135, and Y.

In contrast with the overall lower incidence rates, the CFR for 1989–1998 was 16.6% for New York City, compared to 9.3% for the rest of the United States (*6*). Possible explanations for this finding include differential reporting of severe cases, presence of virulent clones in the population, and timely access of medical care. Additionally, not all public health jurisdictions include probable cases in their surveillance reports to the Centers for Disease Control and Prevention, raising the possibility that the national number of deaths is low because of underreporting of culture-negative fatal cases. A small proportion of cases (5%) in the New York City surveillance database met the definition for probable cases, suggesting that such cases may be underreported; however, no statistically significant difference existed in deaths by case status (confirmed CFR = 16.7%; probable CFR = 21.1%; chi square =0.46; p=0.50). This proportion of probable cases in New York City is comparable to that found in a review of meningococcal disease in New England for 1993–1998, where 4% of the cases met the probable case definition and the CFR was 10% (*14*). The proportion of probable cases and CFR for meningococcal disease in New Jersey in 1990–2000 were 10.5% and 11.6%, respectively (unpub. data, New Jersey Department of Health and Senior Services, Infectious and Zoonotic Diseases Program). An assessment of meningococcal surveillance in New York State (excluding New York City) found delays but relatively complete reporting (*15*). The exceptionally high death rate in 1999 prompted a closer examination of these data. Median age for the year was 35, higher than median age for any other year cohort and significantly higher than median age for all other years (median age excluding 1999 = 21; p=0.013). The proportion of group Y disease was 41%, which also differed significantly from the years excluding 1999 (chi square = 19.0, p<0.01). Further epidemiologic investigation, including molecular typing, is necessary to explain the excess meningococcal deaths in New York City.

### Limitations

A limitation of surveillance-based studies is the bias introduced by underreporting. However, because of the severity of the disease and the need for intravenous antibiotic treatment, most meningococcal disease case-patients are hospitalized, and the local health department is usually notified in order to track down close contacts and ensure that they receive antibiotic prophylaxis. A study assessing the completeness of the New York State surveillance system for meningococcal disease by using hospital discharge data as the basis for comparison showed that 93% of estimated cases in hospitalized patients in 1991 were properly reported (*15*). Hospital practices, such as antibiotic administration before acquisition of cultures, might render samples from case-patients culture-negative; however, no evidence suggests that a change in such practice has occurred in the study interval.

Because our inclusion criteria required a positive bacterial culture, positive cerebrospinal fluid antigen test, or purpura fulminansis, cases that were culture-negative where antigen testing was not available or nonmeningitis cases without purpura fulminans might have been missed. The use of these inclusion criteria was important to ensure the validity of the study and comparability to other jurisdictions but could have slightly inflated the CFR if fatal cases tend to be reported more often to the health departments.

Another limitation was the large proportion of missing information for outcome (22.1%) that may have underestimated the CFR. We minimized this problem by performing death certificate searches using multiple search criteria.

Approximately three quarters of the New York City isolates during the study period were identified by serogroup; this proportion was similar to that observed in other surveillance-based studies conducted in the United States (*2*,*3*,*9*). Assuming that the lack of serogroup information for a proportion of the cases was not related to problems in identifying any specific serogroup (i.e., independent from serogroup), bias was unlikely to have been responsible for the observed trends in serogroup.

The time-trend analysis performed in this study assessed the presence of epidemiologic trends during the 1990s but not the factors responsible for them. Therefore, our study was important in identifying trends, but further studies need to be conducted to test specific hypotheses about the factors responsible for them.

National surveillance data used for comparison of rates and CFR were based on the same case definitions as used in our study; however, not all jurisdictions follow these definitions. For example, New York State Department of Health excludes probable cases, and this variation in surveillance methodology may affect the national CFR used for comparison.

## Conclusions

In New York City, during the period from 1989 to 2000, the overall incidence rates of meningococcal disease decreased. This reduction was more evident in the younger age groups, and therefore the median age of patients with meningococcal disease increased. Independent of the changes in the age distribution, the proportion of cases caused by serogroup Y increased and those caused by serogroup B decreased. The CFR did not change significantly throughout the study period and is higher than national figures. The incidence of serogroup B infections has dramatically declined. Evidence suggests that this decline may be the unintended result of *H. influenzae* type b vaccine use that incorporates the meningococcus serogroup B outer membrane protein. The implications of this finding require further research because currently no available vaccine or satisfactory method exists for controlling outbreaks from serogroup B.

Understanding trends in meningococcal disease epidemiology is important in redefining appropriate measures of control and prevention. The identification of groups at high risk and the distribution of prevailing meningococcal serogroups will be critical in future decisions and recommendations regarding the nonepidemic use of meningococcal vaccine.
